# Glycoproteomics: Making the Study of the Most Structurally Diverse and Most Abundant Post-Translational Modifications More Accessible to the Scientific Community

**DOI:** 10.1016/j.mcpro.2021.100086

**Published:** 2021-06-03

**Authors:** Gerald W. Hart, Lance Wells

**Affiliations:** Complex Carbohydrate Research Center, University of Georgia, Athens, Georgia, USA

In 2013, we served as guest editors for the Glycomics special issue where the Athens guidelines for glycomic analyses were first revealed that have since been adopted by multiple journals (1). At that moment in time, we could not have easily conceived of a special issue dedicated to glycoproteomics. However, just 8 years later, we find ourselves presenting a special issue of *Molecular & Cellular Proteomics* on Glycoproteomics that introduces the reader to the recent explosion in front-end enrichment methods, analytical approaches, and back-end software solutions dedicated to glycoproteomics. This special issue includes nine review articles and nine research articles that introduce the reader to the exciting, challenging, and rapidly evolving field of glycoproteomics, which is so highly dependent on MS.

Glycoproteomics has been front and center this past year as scientists tackled the COVID-19 pandemic, given that the spike glycoprotein of SARS-CoV-2, which is often the basis for antibody and vaccine therapeutics, is a trimer glycoprotein consisting of 66 occupied *N*-linked glycosylation sites, and that the host receptor, ACE2, is also heavily glycosylated (2, 3). Thus, an issue devoted to understanding this heterogenous class of post-translational modifications seems very timely. The number of various glycan moieties that can modify proteins easily surpasses the sum of all other post-translational modifications combined, and the majority of all expressed mammalian proteins (secreted, membrane bound, and intracellular) are glycosylated (4). Thus, it is essential to be able to characterize these challenging biomolecules to better understand the fundamental roles that glycoconjugates play in nearly all aspects of physiology and pathophysiology.

A review by West *et al.* (5) gives us an intertaxa evolutionary perspective on glycomics, glycoproteomics, and glycogenomics. Riley *et al.* (6) provide a pragmatic guide to the plethora of enrichment strategies that exist for glycoproteins, while a review by Maynard and Chalkley (7) focuses specifically on enrichment and assignment of *O*-GlcNAc sites on proteins. Research articles by Kurz *et al.* (8) and Blazev *et al.* (9) both illustrate approaches for detailed glycomic analyses that can facilitate improved glycoproteomics. McDowell *et al.* (10) introduce us to imaging MS and lectin analyses for *N*-linked glycans applied to pancreatic cancer tissues, while Chen *et al.* (11) utilize enrichment combined with intact glycopeptide analyses to look at changes in glycoproteins of cerebral spinal fluid in Alzheimer’s disease. The research article by Martinez *et al.* (12) also exemplifies the utility of enrichment and detection methods for defining the O-GlcNAc transferase interactome.

Ye and Vakhrushev (13) tackle the analytical approach of data-independent acquisition for glycoproteomics and Pegg *et al.* (14) implement the data-independent acquisition approach for the analyses of glycoproteins in sparkling wine. Pepi *et al.* (15) introduce us to the challenging class of glycosaminoglycans–proteoglycans, and Persson *et al.* (16) demonstrate the power of domain mapping of glycosaminoglycans for structural characterization of proteoglycans.

Two reviews, by Cao *et al.* (17) and Delafield and Li (18), discuss recent advances in data analysis for glycans, intact glycopeptides, and quantification methods. The reviews by Caval *et al.* (19), and by Hackett and Zaia (20), tackle the challenging problem of describing the inherent heterogeneity of glycoproteins and in calculating the similarities between glycoproteins. Finally, Roushan *et al.* (21) illustrate the power of tools of multiple data analyses for glycoproteomics.

In this special issue, we have attempted to capture many of the recent developments in enrichment and labeling strategies, MS-based interrogation approaches, and data analysis platforms for glycoproteins that have evolved over the last decade. Although multiple challenges still exist, the existing and continuing technological advancements in glycoproteomics are making the study of glycoproteins more amenable to the scientific community at large.

## Conflict of interest

The authors declare no competing interests.
